# Under one roof? Left‐behind children's perspectives in negotiating relationships with absent and return‐migrant parents

**DOI:** 10.1002/psp.2151

**Published:** 2018-04-15

**Authors:** Theodora Lam, Brenda S.A. Yeoh

**Affiliations:** ^1^ Asia Research Institute National University of Singapore Singapore; ^2^ Department of Geography and Asia Research Institute National University of Singapore Singapore

**Keywords:** children's agency, left‐behind children, long‐distance communication, return‐migrants, Southeast Asia, temporalities

## Abstract

Children—whether left behind or as migrants—have remained largely invisible in Southeast Asian migration scholarship. Their experiences and perspectives on migration, as well as how they demonstrate agency within the limits of culturally/socially constructed childhoods influenced by a “hybridisation” of global and local conditions, are often overlooked in favour of adults'. This article addresses this research lacuna by focusing attention on how left‐behind Indonesian and Filipino children between 9 and 11 years of age engage and react to the changes in their everyday lives brought about by both parental migration and parental return. Using both quantitative and qualitative data collected from a larger study on child health and migrant parents in Southeast Asia with return‐migrants, left‐behind carers, and children, this article highlights the experiences of left‐behind children by revealing their agency and creativity in managing changes in their daily lives due to the frequent and transient comings and goings of one or both parents.

## 1 INTRODUCTION

Transnational familyhood as a social formation sustained through cultivating multifaceted social and emotional ties across borders is characterised by the frequent physical comings and goings of key family members (Basch, Glick Schiller, & Blanc‐Szanton, 1994; Bryceson & Vuorela, 2002; Sørensen & Vammen, 2014). Although much attention has been paid to the formation, maintenance, and practices of transnational families, the research focus has been distributed unevenly in favour of adults'—both migrant and left‐behind members'—perspectives thus far, with less consideration of children's experiences (exceptions include Dreby, 2007; Graham, Jordan, Yeoh, Lam, & Asis, 2012; Mazzucato & Cebotari, 2017). To address this gap, recent literature has begun encouraging a more “children‐inclusive approach” to studying transnational families. Nonetheless, more can still be learnt about “children's varying roles in migratory processes” (Tyrrell & Kallis, 2017, p. 329) and how they manage changes in their everyday lives when parents migrate and return.

In this vein, this article explores the reactions of children through shifting familial circumstances due to the migration and return of their parent(s) over time. Acknowledging that children's agency, ability, and consequential actions may change over their life course even as family circumstances vary, it highlights how Indonesian and Filipino left‐behind school‐aged children understand, engage, and react to the effects of their parents' movements in their everyday lives. It considers the strategies they employ to maintaining a transnational relationship with their migrant parents and the adjustments they undergo when their parents return. Pivotal to these explorations is the attempt to highlight and better understand children's agency in navigating the increasing influence of parental migration in the everyday processes of securing household provisioning and reproduction in the developing economies of Southeast Asia.

The article continues with a brief overview of the importance of a child‐centred focus in migration studies before turning to the context and methodological underpinnings of this particular study. To examine how the various forms of children's agency at different ages manifest at distinct varying points in the migration process, the article highlights two transitional “times of migration” (Cwerner, 2001): first, how left‐behind children manage parental absence in relation to the maintenance of long‐distance relationships with their migrant parents; and second, how they negotiate physical (re)contact and renewed relationships when their once‐absent parents return home. Overall, the article considers how children demonstrate agency in maintaining familial ties within a transnational household and how they contribute to (re)building family rhythms as family morphologies shift over their life course and at different stages of the migration process.

## 2 CHILDREN'S AGENCY OVER TIME AND DIFFERING CONTEXTS

An important approach to furthering the understanding of children's agency is to centre on the “experiences, narratives and stories of migrants and young people” within the growing field of migration research (Kallio & Bartos, 2017; McKinney, 2014, p. 5). Youths and children have distinct, perhaps even alternative, experiences, meanings, and interactions within the processes of migration that are often overlooked (Bushin, 2009; McKinney, 2014). A retelling of their migration stories provides the gateway to learning about how young people's (including children's) identities, ambivalence, and liminality are shaped in relation to their experiences of significant migration events within specific familial contexts in today's neoliberal times of quickened but uneven mobility across borders (McKinney, 2014).

Overall, a child‐centred focus in migration studies helps move traditional depictions of children—whether migrants or left‐behind members—as “baggage” to portraying them as effective migration agents with the ability to shape and influence their fellow family members' lives as well as offer support and help in the migration process (Chiu & Choi, 2018; Dobson, 2009; Huijsmans, 2018; Ní Laoire, Carpena‐Méndez, Tyrrell, & White, 2010; Orellana, Thorne, Chee, & Lam, 2001). Given that children's agency develops largely through their interactions with their family members within a context affected by a “hybridisation” of global and local conditions (van Nijnatten, 2013; Vandenbroeck & Bouverne‐de Bie, 2006), it is especially important to study children of migrant families—including those left behind by migrant parents—within the site of the family where their familial interactions may often be distant, intermittent, disrupted, and/or impeded and where their ensuing situated reactions may also be varied.

Children's “agency” or “capacity to affect things”—not merely in terms of resistance—is not uniform but dependent on structures of power and inequality and must be considered in terms of intentionality, cultural constructions, and power (Ortner, 2006, p. 137). Though children do have the intention and capacity to perform consequence‐bearing actions, their relative position to other persons or conditions can influence their ability to effect any significant change, thus rendering them comparatively “passive” at certain moments (James & James, 2004).^1^ At the same time, although they are not “necessarily powerful political actors who can take a major part in shaping their own histories” (James & James, 2004, p. 24), their acting capacity and passivity may change progressively in noticeability and intensity over their own life course and shifting circumstances as they acquire more “extensions that affect individuals' capacities to act” (Blazek, 2016, p. 226). Even as children's agency and lives are affected by various social, cultural, and economic conditions in which they are embedded, their situated (re)actions—whether in compliance, conciliation, or resistance—are concurrently reliant on their individual competencies in discernment, perception, management, rationalisation, and problem‐solving within the confines of the very context in which they live (Choi, Yeoh, & Lam, 2018).

Temporality or temporal orientations are equally fundamental aspects of agency insofar as ones' interactions and responses to situational exigencies matter (Hitlin & Elder, 2007). Time is especially a critical component when studying children's agency within the migration context given its important relationship to both children's developmental growth and life course as well as migration processes (Carling, Menjívar, & Schmalzbauer, 2012; Elder, 1998). First, children's developmental stages vary considerably through their infancy and adolescence, and children in late middle childhood (generally ages 8 to 11) would particularly be developing increased abilities in thinking, reasoning, understanding, and expression (Feldman, 2010; Meggitt, 2012; Santrock, 2007). In the same vein, children would interact, react, and be affected differently at the various conspicuous times of migration (and/or when being left‐behind)—for instance, times of adjustment immediately after migration (strange, heteronomous, and asynchronous), increasing ambivalence as migration experience develops (remembered, collage, and liminal) and long‐term temporal outlook of the migration experience (nomadic and diasporic) as proposed by Cwerner (2001).

At the same time, space and place also matter in the children's process of maturing and development. Recognising that childhood is both a social and *spatial* construction, there are demonstrably “pervasive spatial discourses in societies that work to contain, channel or prevent children's agency” whether in their places at home and/or neighbourhood or their relative “place/position” as children in the household hierarchy (Nairn & Kraftl, 2016, p. 4). The shaping of children's identity and agency are very much processes that are “enmeshed in relations that comprise not only individual interactions at the micro level but also larger cultural imaginaries linked to neighbourhoods and society,” as well as global processes (Raffo, 2011, p. 4). On the one hand, spaces/places are continually (re)made through human actions and emotions such as routines, habits, and normalised behaviour thus compelling people (including children) to behave in particular ways. Yet, spaces/places do not just constrain actions and practices, and it is equally important to examine children's experiences and expressions of agency, creativity, or subversion in such adult‐defined places to better comprehend intergenerational relations and tensions occurring within them (Nairn & Kraftl, 2016).

This article is thus concerned with how left‐behind children's actions and/or nonactions are shaping their worlds even as they continually develop within a migrant household. It recognises both children's capacity and incapacity in relation to their shifting environment and avoids making excessive assumptions on their behalf to prevent confining them to any particular position from the outset. Children's agency is examined within their personal contexts and familial relationships as left‐behind children of migrant parents and at “the micro level of peer interactions” influenced by global processes across a timeframe characterised by the rhythms and ruptures of their parents' (sometimes multiple, often indefinite) departures and returns (Vandenbroeck & Bouverne‐de Bie, 2006, p. 128).

Here, children's situated agency is brought to the fore through an exploratory examination of Indonesian and Filipino left‐behind children's stories. Childhood experiences are notably diverse across and within both countries, but the lives of the left‐behind Indonesian and Filipino children in this article are joined by a common thread of growing up within a prevalent migration context that is influenced by a host of factors including gender (of migrants, carers, and children), length of migration, and destinations. Although we are increasingly gaining insights into the mixed impact of parental migration on the citizenship (see Butt & Ball, 2018) and developmental aspects (such as behaviours, education, mental and physical health, and relationships) of Indonesian and Filipino childhoods through a growing number of studies (examples include Asis, 2006; Battistella & Conaco, 1998; Graham & Jordan, 2011; Graham et al., 2012; Parreñas, 2005; Sukamdi & Wattie, 2013), it is nonetheless still difficult to derive a comprehensive understanding of left‐behind childhoods. Existing studies investigating the impact of parental migration on specific aspects of children's well‐being seldom include children's perspectives and inadvertently end up portraying children as relatively passive vessels waiting to receive the effects of the adults' actions (exceptions include Episcopal Commission for the Pastoral Care of Migrants and Itinerant People/Apostleship of the Sea‐Manila, Scalabrini Migration Center, & Overseas Workers Welfare Administration, 2004; Parreñas, 2005).

In surveying the broader political and economic context in which Southeast Asian children are growing up, scholars (see Ball, 1997; Battistella & Asis, 2013; Silvey, 2004; Yeoh, Platt, Khoo, Lam, & Baey, 2017) have highlighted the importance of transnational migration as an established economic livelihood strategy used by many families living in the Indonesian and Filipino archipelagos since the 1980s and 1970s, respectively. Although both countries' colonial heritage—Indonesia as a former Dutch colony and the Philippines as a former colony of Spain and subsequently the United States—provides a significant backcloth influencing the migratory propensity of their respective populations, deeper integration of the region into the global world order over the last two decades has led to uneven economic growth and development, which has in turn transformed contemporary migration opportunities and destinations.

Of key significance amidst rapid change is both Southeast Asian countries' incorporation into global care chains as source countries supplying low‐waged female migrant workers to the domestic and care work sectors of the more developed economies in Asia and the Middle East. The governments of both countries have facilitated the international export of women—including mothers—as part of national development goals to reduce domestic unemployment and poverty through growing remittance inflows. The resultant feminisation of labour migration in the region as more women become transnational breadwinners to support their families has far‐reaching consequences on household transitions and childcare (Graham et al., 2012). Given the prevailing temporary migration regime in the region where individuals migrate to destination countries as contract labour on time‐limited entry visas, children of migrant parents are left at source, spending part or even all of their growing years in their overseas parent(s)'—often the mother—absence. Although no official figures are available, estimations postulate that around 1 million Indonesian children and some 3 to 6 or even 9 million Filipino children are living apart from their parents due to migration (Bryant, 2005; Parreñas, 2005; Reyes, 2008).

Even as Indonesian and Filipino children are potentially reaping the economic, educational, and material benefits from migration (Asis, 2006; Khoo et al., 2014), they are also coping with living prolonged periods without one or both parents under the same roof. Despite rising remittance revenues, there is no corresponding increase in development and/or economic opportunities at home, and underdevelopment, underemployment, and unemployment persist (Battistella & Asis, 2013; IOM, 2010). Hence, temporary migration has become entrenched as a commonly used household advancement strategy, and the transnational family formation looks set to continue for generations to come.

## 3 METHODOLOGY

Drawing on both quantitative and qualitative data from Child Health and Migrant Parents in Southeast Asia (CHAMPSEA), a larger mixed‐method study investigating the impacts of parental migration on various aspects of children's well‐being and health in Southeast Asia (see Graham & Yeoh, 2013 for more details), this article attempts to achieve a deeper understanding of left‐behind children's experiences in the migration process through a triangulation of three to four viewpoints collected at different periods. First, responsible adults, primary carers (who may also be responsible adults), and children aged 9 to 11 from roughly equal proportions of transnational and nonmigrant households in East and West Java, Indonesia (*n* = 513 households), and Laguna and Bulacan in the Philippines (*n* = 500 households) were surveyed in 2008. Children's ages were first captured during the survey, and they have grown over the course of the study.

Among these surveyed households, subsequent in‐depth interviews were conducted with 52 children, 74 carers, and 20 return‐migrants from East Java and Laguna in two stages between 2009 and 2012. The interviews—supplemented by survey data—revealed, through the voices of both adults and children, insights into the everyday household dynamics in migrant and nonmigrant families at different phases of the migration process—during migration and after return—and over a segment of the children's life course. This article excluded surveyed households with children aged 3 to 5 and used pseudonyms to protect respondents' identity.

To understand the impacts of migration on children under 12, the study surveyed and interviewed children ages 9 to 11 to capture preadolescent views at the tail end of “childhood.” As mentioned earlier, children in this stage have higher awareness and are able to express themselves better than younger children but still have limited capacity to act in comparison with adolescents. Interviews with children adhered closely to approved ethics guidelines and adopted ice‐breaking and confidence‐building activities such as the “Protection Umbrella” (adapted from Beazley, Bessell, Ennew, & Waterson, 2005). Interviewers were mindful, sensitive, and observant of children's moods, allowing them to talk freely or remain silent as they wished. They did not always/completely adhere to the aide memoire and chose to sacrifice certain questions or probes in favour of child‐driven answers/interest.

Interviews were mainly conducted in native languages within adults' eyesight but out of earshot, transcribed and translated into English, and carefully analysed by researchers against the contextual backdrop and observations of children's emotions detailed in field notes using NVivo. The coding process started with some broader thematic nodes based on the interview guide. New nodes arising from children's responses were developed from a thorough reading of the transcripts, and all nodes subsequently reviewed to find common themes and grouped under overarching tree nodes. Similar nodes were applied to adult interview transcripts in order to triangulate the data. Although the codes helped us organised the data more systematically, analysing children's interview transcripts went beyond observing common themes to take into account the specificities of emotions that were expressed or restrained, the occurrence of inaudible words and silences, and the detailed contextual information obtained from field notes.

## 4 CHILDREN'S AGENCY IN MANAGING LONG‐DISTANCE COMMUNICATIONS

Notwithstanding their young age, the majority of the children surveyed in the study (78.2%) were aware of the reasons behind their parent's long‐term absence from the household, with more than half (51.9%) indicating that parental migration was triggered primarily for their education. At the same time, although most expressed feeling sad about their parent's migration through the surveys (71.0%) and interviews, they were also able to recount the benefits—particularly material well‐being—of having a migrant parent during the interviews. Despite having little say in their parents' migration decisions and feelings of sadness in the immediate aftermath of their parents' departure, most of the left‐behind children over time displayed considerable fortitude in coping with parental absence. This is demonstrated by the way children adapted to and carried out relatively stable daily routines with the usual mix of school, household chores, prayers, and play, echoing the finding of Episcopal Commission for the Pastoral Care of Migrants and Itinerant People/Apostleship of the Sea‐Manila, Scalabrini Migration Center, & Overseas Workers Welfare Administration (2004) where parental migration did not affect children's socialisation or the education and development of critical values, responsibilities, and spirituality. Shirot (11, Indonesian), for example, grew emotional when missing and thinking of his mother during the interview, but he persevered:

I was quite unhappy [because mother] left me again [for Saudi Arabia]. … [I want] both my parents [to care for me] because I feel that my family is incomplete. [Still,] I wake up by myself. I take a bath by myself. I do [homework] myself. Sometimes, I ask someone if I have some difficulties. … I examine the rice field and do the cooking … many things [with my father]. [I'm] second or third [rank in class] … [I make my mother proud] because I never do bad things.

A part of negotiating parental absence in their daily lives requires left‐behind children (compared with those from nonmigrant households) to incorporate long‐distance communication links with their migrant parents into their routines. In fact, the larger proportion of left‐behind children (66.6%) surveyed reported having weekly contact using communication technologies with their migrant parents, ranging from daily (13.1%) to a few times per week (25.2%) to at least once a week (28.3%). Notwithstanding such seemingly frequent contact nearly three quarters of the children have with their migrant parent(s), closer analysis of the interviews also revealed the limits within which children could exercise agency due to their uneven access to telecommunication technologies (similar to findings from Ansell, 2009; Hoang & Yeoh, 2015; Parreñas, 2014; Vertovec, 2004).

Apart from having little control over the telecommunication infrastructures available within their communities, many children were also constrained within their own homes in terms of their means of initiating or accessing the necessary tools to contact their parents directly. Similar to other studies (see Hoang & Yeoh, 2015; Parreñas, 2014), it was usual to hear many of the children sharing during interviews their inability to initiate communication with their migrant parents as and when they wish. For most left‐behind children, contacting one's parent was often not as simple as Pauline (9, Filipino), who had ready easy access to a cell phone, described, “I look for her [migrant‐mother's] number and I press the numbers so I can talk to her.” Instead, many could do “nothing” but “wait” for their overseas parents to call first because they either do not have the capacity (often economic), permission, or access to phones to make calls. The length of this waiting period is also affected by other factors such as time difference and migrants' respective working and living situations that may further restrict telecommunications access (e.g., migrants in occupations such as live‐in domestic work were unable or only able to call at times when children would have gone to bed; Graham et al., 2012). For some of the children, this “waiting” was built into a regular schedule implemented by the adult members of the household, and where they were willing to accept as they did not feel the urge or need to modify communication patterns. Aware that their parents would call at fixed days/times, Gladys (10, Filipino) was contented to do nothing to initiate communication “because we chat every week, I just tell him [father] what I want to say then,” whereas Candice (11, Filipino) would do “nothing, I just wait for Fridays [when migrant‐father calls].” Children's display of limited agency in this regard further affirms the view that sustaining transnational family ties is in many ways cast predominantly as an adult‐controlled set of tasks.

Children's age, level of maturity, and “readiness” were some of the reasons adult carers and parents gave for denying them access to a mobile phone and further retaining parental control over children's autonomy. Compared with older siblings, preprimary and primary school‐aged children under 12 were often still regarded as too young to own their personal mobile phone. Thus, many of the children interviewed did not own a personal mobile phone and needed to borrow one from other family members to gain access. Oji (10, Indonesian) recounted her negotiations with her migrant‐mother over phone ownership, “Mom, would you buy me a cell phone? [*Ibu* answers], ‘Not now, Oji. Later, if you're old enough. It's better if you study hard now’.” To which, Oji appeared to acquiesce in responding “alright mom!” However, there were clear indications that children aspired to possess their own mobile phones, and some became less accepting of this restriction (Graham et al., 2012). Rather than heed her mother's instructions to wait until she was older and had demonstrated responsibility by studying harder, children such as Oji exercised both persistence and strategy in insisting on their right to mobile phone ownership. Despite seemingly agreeing with her mother, Oji turned to persuading her left‐behind father instead, “Dad, would you buy me a cell phone? Most of my friends already have a cell phone!” In fact, several children interviewed had apparently succeeded in their persuasion and shared that they were eagerly awaiting the migrant parents' return and fulfilment of their promise to gift them a mobile phone. Although more of the Filipino children interviewed owned personal mobile phones as compared with their Indonesian peers, children's (whether Indonesian or Filipino) freedom in using their mobile phones was still dependant on adult members' generosity in granting them phone credits (Graham et al., 2012). Thus, children's access (and control over this access) to communication technologies varied across country and age, and fluctuated in relation to controls imposed by adults.

Children's personal capacity at particular points in time also influenced their access to communication technologies and, in turn, their ability to contact their parents. Ten‐year‐old Pani (Indonesian) claimed that he “can't write” and relied on his older brother to type text messages on the mobile phone to his migrant‐father for him. However, such impediments—as with their access to telecommunication devices—would change over time as technologies develop and children learn new skills to facilitate their communication styles with their absent parents. Claire (10, Filipino), for example, shared that she contacted her migrant‐mother only by phone previously because “I didn't know how to use the computer and internet at that time.”

Among the children interviewed, some have progressed toward gaining semi‐autonomy in managing long‐distance communications with their parents. A few could convey their wish to connect with their parents by first “sending a text,” personally making a “missed call” to the parent, or relying on an intermediary such as their left‐behind parent or older sibling to make contact on their behalf. Wulansari (9, Indonesian) was further mindful about the costs involved, “It's expensive, so I never call him [migrant‐father]. I give him a missed call [and he would call me back].” These left‐behind children have acquired the adeptness and strategies of cultivating long‐distance communication with their migrant parents through a set of agreed routine practices—of which they have partial control—working toward a relationship built on proximity and instantaneity (Parreñas, 2014). Even if there was still some waiting involved for the migrant's return call, these children could at least independently express and act on their desire to reach their migrant parent.

Although communication is often conducted “to produce intimacy and to nurture the family” (Parreñas, 2014, p. 439), what children say or omit during their conversations further revealed their agency in negotiating long‐distance intimacy with their absent parents and influencing the degree of involvement absent parents had in their daily lives. Conversations could range from outright antagonistic rejections of contact or intimacy to highly intimate moments when children expressed their love, concern, and longing for the absent parent. Some children also expressed empathetic understanding of their migrant parents' circumstances even where they had limited information and only their imagination to go by. They then strived to develop a more positive long‐distance relationship with their migrant parents. Marianne (9, Filipino), for example, felt sad imagining her mother's life overseas and tried hard to be more understanding and loving toward her during their conversations: “I was sad because I thought of mommy having a difficult time there. … that it was difficult to work there. [So to comfort her] I told her that I love her.” Similarly, Kelvin (11, Filipino) would reflect on how his improved life was made possible by his mother's hard work and would make up with her immediately whenever he became upset with her:

… I've been mad at her before when she was abroad because of some broken promises … The other day when I awake, I always text her I'm sorry for being mad at her. When I lay in bed, I kept thinking about … our situation here in the Philippines. That's why when I realised, I ask forgiveness the next morning. No, [I don't think she's having a good life in Dubai] because it's hard … even me in this small house, [I] may take a long time to clean this house so especially my mom [working in] a big house. It's ok for me [even if she's looking after other children instead of me] … that's my mother's job, I can't complain about that.

A fuller view of children's agency became particularly evident in the way some children, akin to adults, restrained themselves in consideration of their migrant parents by withholding critical information to avoid burdening or worrying them. Despite wanting desperately to own a mobile phone, Bulan (10, Indonesian) did not want to ask her migrant‐mother for one, “Aunt knows [I want a cell phone with camera] but I don't feel it appropriate [to tell my mother]. Yes, [because I feel sorry for her].” In another account, Justin (9, Filipino) displayed consideration for his father as well as other family members by regulating his telephone conversations, “I don't tell him [migrant‐father] about my life here [anymore]. He might run out of prepaid cell phone credits [and] it's [the conversation's] over!” Though Justin loved telling his father stories about himself, he showed growing maturity and increasing familiarity with navigating the limitations of long‐distance communication by restricting himself so that his father would have more opportunities to share about his overseas life instead.

In other cases, children's already distant relationship with migrant parents placed limits on their conversations, hindering them from growing closer. According to Abadi (42, Indonesian), a father‐carer whose wife has worked abroad intermittently for almost 6 years, Awuna (11, Indonesian) “is rather shy with her mother. Therefore, she just shares some stories if she wants to ask something or asked to be sent something such as a mobile phone. However, she has never shared her feelings to her mother … She does not know what to talk about if the mother does not ask her first.” Although Awuna was clearly aware that a conversation with her migrant‐mother was necessary to get what she wanted, she remained reticent in sharing more intimate details that may further improve their relationship.

Although the availability of communication channels had paved the way for mediated forms of intimacy over distance, not all children were willing to engage their absent parents in this way. Some like Gladys would refuse to elaborate when asked by her migrant‐father for details of everyday life events. Others, particularly those whose parents left when they were very young, felt alienated from their migrant parents and were, at least initially, reluctant to converse with them. Still, others expressed a sense of resistance by refusing contact to avoid being reprimanded. Referring to her daughter Isabelle (11, Filipino) and son, mother‐carer Marvie (42, Filipino) said,

[She refuses to talk to her migrant‐father] when she made a mistake. She knows [he will get angry with her]. She has the same attitude as her older brother. When they know they did something wrong and their father would call, they would refuse to talk to him. Sometimes he would call their personal cellular phones and they would turn it off.

Isabelle and her brother were clearly proficient at manipulating communication technologies to their own advantage, knowing that they could simply “turn it off” to avoid a scolding and turning the phone back on again when the coast clears.

Generally, the left‐behind children in this study had little say over their parents' migration decisions, which parent migrated and for how long. Yet, with varying degrees of control, they were able to express some agency in negotiating long‐distance relationships with their absent parents. Many were particularly limited in their efforts to initiate contact or access communication technologies. However, their agency becomes more apparent in their management of long‐distance relationships through their choice of conversation topics and disclosure of information. Most of the children were not passive in trying to gain more control over communication channels, particularly as they grow older. Rather, they exhibited an eagerness to gain some level of autonomy, by being vocal or strategic in requesting for mobile phones and learning new skills. Children's attempts to control conversational exchange and information flow further revealed their growing maturity and sensibility in managing transnational relationships, throwing some light on the roles they played in furthering transnational family projects.

## 5 WHEN PARENTS RETURN TO THE FOLD: (RE)BUILDING THE FAMILY RHYTHM

Growing up in the absence of one or both parents for long stretches of time necessitated a certain amount of adjustment for left‐behind children when migrant parents returned to the household. Alongside personal developmental changes, children had to confront changes in the household and care arrangements when the migrant returned, whether temporarily or permanently, and adjust their behaviour—reflecting varying degrees of compliance, compromise, or resistance—accordingly. Although most children were usually told to anticipate being reunited with their absent parents, they had to accommodate the “sudden” physical presence of a person in their daily routines who had previously been an “absent presence” for a large part of their formative lives. Their initial reactions thus varied considerably, ranging from avoidance (“hiding from” return‐migrant) to being overly sticky (“always together”), and continuously changing over the adjustment period. For example, Paku (11, Indonesian) felt awkward seeing his mother again upon her return, “I said nothing [when I first saw *ibu* on her return]. I did not [hug or kiss her]. I did not want [her to kiss and hug me]. I was shy.” His mother Sherly (37, Indonesian) continued, “He was shy at first. When he came home from school, he would usually go this way, but he went that way instead [to avoid me].” On the contrary, Veronica (34, Filipino) shared her close relationship with Jade (10, Filipino), “We are good. We are always together, close to each other. I hug her, kiss her.”

Rather than merely waiting for return‐parents to make the effort to reconnect with the family, many children were also instrumental in synchronising the return‐migrant into the rhythms of an already established family life. This was the case even when some of these changes to the family rhythm were not particularly welcomed by the children. A commonly encountered change that many children described was the enforcement of a stricter discipline regime by the return‐parent, particularly mothers. Free‐spirited Claire woefully complained she was “no longer free” to go wherever or do whatever she wanted now that her mother was home. Besides losing much liberty to move around the neighbourhood freely now that they had to succumb to an additional pair of watchful eyes, children also reported having to get used to newly enforced restrictions on playing computer games, tighter curfews, and more intense scrutiny on personal hygiene. Indonesian children such as Victori (aged 10) experienced a reduction in playtime in favour of increased prayer time when their mothers returned. Most children also found themselves having to focus more on study sessions, and some said that they performed better academically when their migrant parents were home.

Like Claire, many children did not relish the stricter discipline and tighter restrictions imposed on them after the migrant's long absence. Nonetheless, as Claire's return‐mother Jenna (31, Filipino) attested, the children did not protest too much and were generally obedient in order to facilitate a closer relationship with the return‐parent. Although Marianne was unhappy with her return‐mother's strict and controlling ways, she said she “never complained” and did what she was told. She also expressed wanting her mother around at home, even as her mother prepared for yet another migration stint. Although children's obedience could be viewed as their passive capitulation to a more circumscribed regime, the children's own accounts suggested that their willing compliance and seeming loss of autonomy were also aligned with the role they played in making space for the return‐migrant in their lives in trying to reintegrate the once‐absent parent into the family rhythm.

Children also discovered that they had to perform more household chores at a higher standard of cleanliness after their mothers returned. Kelvin lamented,

Before when my dad is only the one [here], we only clean our house maybe once a week. But then when my mom was here, we have to clean it every day. So when I heard that classes were about to resume, I was very happy I was not cleaning any more. Sometimes I help when I see my mom cleaning, I go and help because I know she can't do it. But then sometimes when I'm feeling so tired, my mom has to tell me to clean. I sweep the floor, when these windows get dirty I have to wipe them and also those appliances. And then I always wash the dishes at night except when I have homework and also cooking rice.

Although Kelvin had the option of rebelling against his mother's instructions, he chose to comply and. He helped his mother even when he was tired as he was worried about her slipped disc injury sustained while working overseas. He was intent on ensuring that his mother had a relatively relaxing time whilst recuperating at home. Nonetheless, he was still relieved that the start of school had given him a legitimate excuse to stop doing household chores, and therefore avoiding an open confrontation with his mother.

Besides doing what was asked of them, children also played a role in (re)stitching familial relationships now that the migrant has returned. While return‐parents were eager to spend more time reconnecting with their children, children also reciprocated by giving up playtime with their friends to stay close by their parent's side. Children such as Jade and Dumadi (10, Indonesian) also turned more “childish” and reliant in demeanour and, through episodes of “babyish” behaviour, allowed return‐parents to resume their parenting roles, re‐bond with their children, and reconnect to the household web of care. Some of the more mature children acted even more purposively to promote intimate interactions among family members by requesting that the family engaged in joint activities such as cooking, shopping, “hanging out,” or “watching television programmes” together. A few were also conscious of the need for their once‐separated parents to spend some quality time together. Their actions and thoughts were exemplified by Kelvin's sharing,

I don't go out that much anymore like when she's [mother] not here. No, [it's not a sacrifice]. I want to stay at home. … One time we went to Enchanted Kingdom [a theme park] in Santa Rosa. Sometimes we go to SM [the mall]. [Together, doing] those happy things. Bonding. … Right now, I wouldn't like both of them [parents] to migrate because it's not been long since my mother has been home. So I would like them to have some bonding time … It could be for a year or two?

Overall, left‐behind children in the study appeared to adapt well to the changes brought about by the migrant parents' return. By and large, they responded positively to the additional care and supervision from the return‐parents. More importantly, rather than passively adapting to their parent's return, they played an active role alongside return‐migrants in ensuring that the family became “more harmonious, happier” (Yuda, 11, Indonesian) or “livelier” (Satriyo, 11, Indonesian) after the respective return of their mothers and fathers. Many children further recognised that parental migration (and return) had a positive effect on their personal growth. Left‐behind children—by their parents' and own accounts—had generally matured due to the experience over time. Children such as Yuda became more responsible at home: “I sweep the floor, I prepare my siblings' clothes. I help my siblings in taking a bath.” He also told his siblings off when they misbehaved. For Kelvin, his mother's absence reminded him that he should learn independence and start preparing for the day he should leave his parent's nest,

So for me, I also have to learn them [housework]. I have to learn those, so when time comes, I would be able to help. And also I need to learn those because my mother and father will not always be there for me. Because maybe when I go to college, when I go to a far place, school that is far from our house, I have to rent an apartment. So I know how to do those stuff [*sic*].

## 6 LIVING THROUGH RUPTURES AS LEFT‐BEHIND CHILDREN

As a long‐term family project to secure economic well‐being and/or achieve social mobility, transnational labour migration carries considerable foreseen risks. Regardless of the sacrifices made by family members and the efforts to maintain long‐distance contact, migrants' physical return does not always contribute to an immediate or guaranteed (re)“gluing” of familial ties, particularly when ruptures (an intertwining of “continuity and change” amidst peoples' movements) have emerged during the separation leading to members of the family living asynchronous lives/times (Boehm, Hess, Coe, Rae‐Espinoza, & Reynolds, 2011, p.1). In a small minority of cases (under five) in our study, children felt that they were no longer able to cope with the rupturing effects of their migrant parents' absence and grew insistent that they returned. Jade—a special needs child—might not have fully understood the multiple ruptures occurring in her family during her mother's absence. Her older sister had run away from home and became pregnant, her older brother had also dropped out of high school after missing too many classes due to stomach pains (later discovered by the migrant‐mother to be urinary tract infection), and her grandfather fell ill. Jade personally had to stop school as there was no adult to take her there. Realising the cracks showing up in the transnational family, she demanded her migrant‐mother's return, “Come home! Grandfather is sick!”

Children's ability to articulate their expectations of their parents also changed over time and growth. Although Harum (34, Indonesian) did not encounter overt resistance when she first left to work abroad some two and a half years ago, she recounted what her son, Yuda, said to her midway through her contract,

‘Why did you not look after us, but you looked after the other people's children?’ I replied that I am working for our subsistence. He asked me to go home, ‘Mom, come home soon! I want you’. I said, ‘I'll go home soon’. My visa was not finished yet, it also depends on my contract.

As an older child now, Yuda expected *his* mother to be home caring for him and his younger siblings rather than “other people's children.”

Although Yuda was fortunate that his mother acceded to his request and returned home fairly quickly, Arnold (23, Filipino)—who experienced being a left‐behind child for much of his adolescence—recounted that he was unable and/or unwilling to mend the 10‐year gap between himself and his migrant‐mother and migrant‐stepfather.^2^ Now a married man with his own infant child and also primary carer to his younger stepsister, Arnold recounted his experiences of being left behind at 13, shedding insights on potential ruptures in the face of continued parental migration. Describing the hardships he endured when his mother migrated,

It's hard because you need money and that's always insufficient. And because there were some debts left so when someone comes [to demand payment], I'm the one who has to face them … sometimes they [migrant parents] can't send money, so it's hard.

Although he admitted to a deviant childhood,^3^ he remained optimistic about the future: “Now, I'm getting better at it. [I don't regret it!] Of course not, I can't do anything about it, it's there already. [Life was also] joyful because we're always complete [even without his parents].” At the same time, the ruptures of migration had left an indelible mark—for Arnold, his parents no longer featured in his idea of a complete family, and he considered their absence as “normal.” He was so accustomed to their absence that he would deliberately move away from the family home when they returned for temporary breaks as he did not want to “have to adjust [to their presence or departure].” He continued, “whatever I was before [without them], that will be the same.” Arnold's story revealed his struggles as a left‐behind child, forced to become an adult/parent/carer all too soon in the wake of parental migration. In his view, dissociating himself from the comings and goings of his migrant parents had become his best protection against further ruptures in his life.

Upon further probing, Sira (9, Indonesian) also revealed why his relationship with his migrant‐mother was strained, leading to him avoiding her calls, “She's mean. [She gets angry with me] Because I don't finish eating my meal. She asks me to finish my meal. She pinches me … my ear. [I'm afraid of my mother] … she's mean.” Avoiding his mother's calls was thus a method adopted by Sira to escape her discipline. His father agreed that the migrant‐mother was strict,

Well, she is … a bit mean when she is at home. If her children make a little mistake, they will be pinched … they fight their mother, something like that … [But when the mother is away] they have never been beaten … it is not permitted.

The estrangement reached a point when his migrant‐mother had to threaten withholding gifts if the children refused her calls, “[The mother said], ‘if you do not wish me to call, do not ask anything from me’, something like that, then the children wish to [talk to her].” Although Sira initially expressed happiness about having his mother home, he could not accept her “mean” or overly harsh way of demonstrating care during her brief visit. He confessed that he did not miss his mother and hoped that she would remain a migrant as long as possible so that he could continue escaping direct contact with her. In some cases, migration amplified existing tensions in the parental care relationships. The happiness of having a parent home might be eclipsed by feelings of resentment over these unresolved tensions.

## 7 CONCLUSION

Although the two neighbouring archipelagos in Southeast Asia have different colonial and religious histories, Indonesia and the Philippines have experienced similar developmental pressures in recent decades and followed a comparable pathway in becoming major migrant‐sending, remittance‐dependent countries. As a result, many children in the two countries are growing up in the absence of one, and sometimes both, parent as overseas work—often in retrogressive occupations such as domestic work (for the women) and construction labour (for the men)—has become a significant means of lifting families out of poverty or achieving socio‐economic mobility. This, however, does not mean that Indonesian and Filipino left‐behind children are passive observers of their parents' migration. Similar to Asis' (2006) findings, children have agency in determining migration outcomes, playing an active role in minding their own well‐being, coping with parental absence, and also keeping the family together. Despite their relative powerlessness in larger migration and communication decisions, left‐behind children from both countries have managed to project their agency situated within the confines of a migrant household in various unique ways, even managing to effect some form of change in their families and fulfilling the conditions of agency proposed by Ortner (2006). To further reiterate James and James' (2004) point, children's agency need not necessarily manifest in “big” ways or result in “major” changes. The results of their actions may also not be immediate but only becoming evident several years down the road. Nonetheless, the interactions between children and the adults do make “a difference—to a relationship, a decision, to the workings of a set of social assumptions or constraints” (Mayall, 2002, p. 21).

Children's situated agency was explored in this article through their home spaces at two distinct “times” in the transnational labour migration process, namely, during the parent's time away and when parents return, and also over time as children grow in maturity. Children had varying roles to play in each migration period, maintaining transnational relationships, reintegrating return‐parents into the familial relationships, and also managing ruptured relationships. Through their actions, children were able to influence the degree an absent parent can remain present in the home in a parallel state of synchrony, as well as the extent a return‐parent can harmoniously synchronise with the established family rhythm. Children's actions may also contribute to either closing or furthering ruptures that have emerged during the parent's absence in the course of migration.

## ENDNOTES

1 Others may view children's passivity as a “diminished form of agency,” purposive, and intentional (Collins & Tymko, 2015, p. 77).

2 Arnold's stepfather migrated to join his mother in Italy after remaining home for 8 years. His birth father was not mentioned during the interview.

3 Though existing studies and anecdotes often hinted that Filipino daughters are left in less favourable circumstances than sons, Arnold's story is reminiscent of left‐behind daughters who would get married and/or pregnant early in order to form their own complete families (Parreñas, 2005; Sampang, 2005). Experiences of family ruptures are thus not gender specific.
